# Expression of the epidermal growth factor receptor family in prostate carcinoma before and during androgen-independence

**DOI:** 10.1038/sj.bjc.6601536

**Published:** 2004-01-20

**Authors:** E Hernes, S D Fosså, Aa Berner, B Otnes, J M Nesland

**Affiliations:** 1 Department of Clinical Cancer Research, The Norwegian Radium Hospital, Montebello, N-0310 Oslo, Norway; 2 Department of Pathology, The Norwegian Radium Hospital HF, Oslo, Norway; 3 Department of Surgery, Bærum Hospital, Bærum, Norway

**Keywords:** prostatic neoplasms/receptor, epidermal growth factor/receptor, erbB-2/receptor, erbB-3

## Abstract

Novel palliative strategies for patients with androgen-independent prostate cancer (AIPC) include targeting the epidermal growth factor receptor (EGFR) family. The aim of the present study was to investigate intrapatient changes of EGFRs during the development of AIPC. In total, 106 symptomatic AIPC patients were identified in whom prostatic biopsies (adenocarcinoma) were available both before the start of androgen deprivation (PRTR biopsy) and after the development of AIPC (AIPC biopsy). All four known subgroups of the EGFR family were determined by immunohistochemistry (IHC): c-erbB-1 (EGFR), c-erbB-2 (HER2/neu), c-erbB-3 (HER3) and c-erbB-4 (HER4). Moderate to strong membrane-specific staining was recorded semiquantitatively (<10% *vs* ⩾10%=IHC stained tumour cells: ‘negative’ *vs* ‘positive’ staining). The medical records were reviewed for clinical variables. During the development of AIPC, intrapatient changes occurred in two opposite directions for each of the four EGFRs: negativity changed to positivity, and vice versa, statistically significant only for the increase of c-erbB-1 expression (*P*=0.001). The c-erbB-2 expression in the AIPC biopsy was associated with a significantly shorter survival from the time of the AIPC biopsy (*P*=0.029). Our results support ongoing therapeutic attempts of EGFR inhibition in subgroups of patients with prostate cancer. Further research is needed to understand the function of EGFRs in this malignancy.

Androgen-independent prostate cancer (AIPC) is defined as biochemical and/or clinical disease progression during androgen deprivation with castration levels of serum testosterone. Patients are in need of palliation, and novel strategies focus on the epidermal growth factor receptor (EGFR) family and related intracellular pathways.

Based on current knowledge, the EGFR family comprises four members; c-erbB-1 (EGFR), c-erbB-2 (HER2/neu), c-erbB-3 (HER3) and c-erbB-4 (HER4) (Olayioye *et al*, 2000;Suo and Nesland, 2002). All members of this family have an extracellular ligand-binding domain, a single transmembrane domain and a cytoplasmatic tyrosine kinase domain, which for c-erbB-3 is nonfunctioning. Binding of ligands to the extracellular domains leads to the formation of homo- and heterodimers. This starts a complex signalling cascade finally resulting in cellular proliferation, prevention of apoptosis and promotion of tumour cell mobility, adhesion and invasion (Barton *et al*, 2001).

Previous observations have demonstrated increased levels of c-erbB-1 immunoreactivity in hormone-independent human prostate cancer cell lines (MacDonald and Habib, 1992; Sherwood *et al*, 1998). In tissue from patients with metastatic AIPC, the c-erbB-1 expression has been reported as high as about 90–100% (Scher *et al*, 1995; Di Lorenzo *et al*, 2002). For c-erbB-2 expression, there have been reports of divergent rates in primary prostate carcinoma (Mellon *et al*, 1992; Kuhn *et al*, 1993; Fossa *et al*, 2002). In xenografts, an upregulation of c-erbB-2 has been found when the tumour becomes androgen-independent (Craft *et al*, 1999). In tumours from patients with AIPC, c-erbB-2 expression is reported from relatively uncommon (Reese, 2001; Savinainen *et al*, 2002) up to about 50–80% (Signoretti *et al*, 2000; Osman *et al*, 2001; Di Lorenzo *et al*, 2002).

To the best of our knowledge, data are lacking from a larger series that compare the expression of all EGFRs for the same patient before and after the development of AIPC. The primary aim of the present study is to fill this gap and, secondarily, to provide information about the prognostic significance of EGFR expression in AIPC patients.

## MATERIALS AND METHODS

From the files of the Department of Pathology, The Norwegian Radium Hospital (NRH), we identified AIPC patients with an adenocarcinoma-positive biopsy from the prostate before the start of androgen deprivation (pretreatment (PRTR) biopsy) and after the development of symptomatic AIPC (AIPC biopsy). The latter histological material was obtained by palliative surgery due to local problems (transurethral resections of the prostate (TUR-P)/transvesical prostatectomy (TV)), or represented a diagnostic biopsy in order to differentiate between a growing prostate cancer and rectal cancer (transrectal/transperineal core biopsies). Patients displaying primary androgen-independence, defined as clinical progression within 3 months after the start of castration therapy, were excluded from the present study. Any concomitant or prior medical and/or surgical treatment was allowed, whereas radiotherapy (RT) to the prostate prior to the AIPC biopsy was a criterion for exclusion. In all, 10 specimens of archival formalin-fixed normal prostate tissue represented a clinical control group.

### Clinical information

The following information was extracted from the medical records:

*Initial diagnosis and treatment*: Date, serum prostate-specific antigen (PSA) level, extent of the disease categorised as local, regional or distant metastases according to the registration routines of the Cancer Registry of Norway (www.kreftregisteret. no), date of start and type of androgen deprivation.

*Clinical course and diagnosis of AIPC*: Date and type of clinical progression (local *vs* distant), date and status of last observation (death or 15 October 2001). For all PRTR and AIPC biopsies, we recorded the date and type of biopsy together with the Gleason score, the latter differentiating between Gleason score 7a (grade 3+4) and Gleason score 7b (grade 4+3) (Lilleby *et al*, 2001).

### Immunohistochemistry

For each eligible patient, the archival paraffin blocks of the PRTR biopsy and of the first available biopsy after diagnosis of AIPC were collected. Serial paraffin sections (5 *μ*m thick) were cut from the blocks with the most representative amount of tumour tissue, as determined in haematoxylin and eosin-stained slides. The newly cut sections were mounted on silane-coated slides and dried for 1 h in 56°C followed by 37°C overnight. The sections were deparaffinised, rehydrated and incubated with 0.3% H_2_O_2_ in methanol for 30 min to block endogenous peroxidase. The pretreatment conditions and the primary antibodies against PSA and the EGFRs are presented in Table 1

**Table 1:** Primary antibodies and conditions

**Antibody against**	**Source**	**Cat. no.**	**Dilution**	**Pretreatment**
PSA	DAKO, CA, USA	M0750	1 : 20	Microwave 2 × 5 min^a^
c-erbB-1	DAKO, CA, USA	M3563	1 : 100^b^	Pronase 10 min
c-erbB-2	Novocastra Laboratories Ltd, Newcastle, UK	NCL-CB11	1 : 200	Microwave 2 × 5 min
c-erbB-3	SantaCruz Biotechnology Inc., CA, USA	SC-415	1 : 50	None
c-erbB-4	SantaCruz Biotechnology Inc., CA, USA	SC-283	1 : 50	Microwave 2 × 5 min

a In 10 mmol/l citrate buffer (pH 6.0).

b Applied overnight.

. Automatic immunostaining with the biotin–streptavidin amplified (B-SA) system (Optimax® Automated Cell Staining System Plus, BioGenex, San Raman, CA, USA) was applied. All series included both a positive control (known positive case) and a negative control in which a nonreacting immunoglobulin of the same subclass had substituted the primary antibody. All controls were satisfactory.

### Scoring procedure

The results of the immunostaining were reviewed by an experienced pathologist (JMN) and scored semiquantitatively as follows: no staining: −(minus); scattered cells to less than 1% tumour cells positive: +; 1 to <10% tumour cells positive: ++; 10–50% tumour cells positive: +++ and >50% cells positive: ++++. Scores of −, + and ++ were grouped together as ‘immunohistochemistry (IHC)-negative’ as opposed to ‘IHC-positive’ findings in the examination of the clinical impact. For the EGFRs, only moderate to strong cell membrane-specific immunostaining was taken into account.

### Statistics

Statistical analyses were performed using the computer-based program SPSS (Statistical Package for Social Sciences) version 10.0. Individual changes of immunostaining after diagnosis of AIPC were assessed by the McNemar test for related samples. The overall survival was estimated by the Kaplan–Meier method from the date of the AIPC biopsy to the last observation/death applying the logrank test to assess statistical significance. *P*-values <0.05 were regarded statistically significant.

## RESULTS

### Patient characteristics

A total of 106 prostate cancer patients (diagnosed 1970–1997) fulfilled the eligibility criteria. The median age at initial diagnosis was 70 years (range: 50–86) (Table 2

**Table 2:** Demographics at diagnosis of prostate cancer

*Age (years)*	70 (50–86)^a^
	
*Extent of the disease*	
Loco-regional	81 (76%)^b^
Distant metastases	24 (23%)
Unknown	1 (1%)
	
*Primary hormone treatment*	
Orchiectomy	56 (53%)
LHRH analogues	33 (31%)
Combined LHRH analogues/antiandrogens	15 (14%)
Oestrogens	2 (2%)
	
*Initial diagnosis to diagnosis of AIPC (months)*	32 (7–167)

a Median (range).

b Number of patients (%).

). In total, 23% of the patients had distant metastases at the time of initial diagnosis. Only five of 34 patients with initially available serum PSA values displayed levels less than 10 *μ*g/l. Androgen deprivation was started within median 2 months after initial diagnosis (range 0–109). After a median time of 32 months (range 7–167) AIPC was diagnosed in all patients. Distant metastases were recorded for 43 patients after the development of AIPC. Of the 105 PRTR biopsies evaluable for Gleason score, 79% were ⩾7b (Table 3

**Table 3:** Histological specimens

	**PRTR biopsy**	**AIPC biopsy**	***P*^a^**
*Type of biopsy*			
TUR-P	26 (25%)	99 (93%)	
TV prostatectomy	7 (7%)	—	
Transrectal/transperineal core biopsy	73 (69%)	7 (7%)	
			
*Gleason score*			
⩽7a (grade 3+4)	21 (20%)	—	
⩾7b (grade 4+3)	84 (79%)	106 (100%)	
Not evaluable	1 (1%)	—	
			
*IHC positivity*			
PSA	88 (83%)	73 (69%)	0.006
c-erbB-1	24 (23%)	46 (43%)	0.001
c-erbB-2	35 (33%)	25 (24%)	0.174
c-erbB-3	15 (14%)	22 (21%)	0.21
c-erbB-4	25 (24%)	31 (29%)	0.43

a McNemar test for related samples.

). All AIPC biopsies were Gleason score ⩾7b.

### Immunostaining

**Figure 1:**
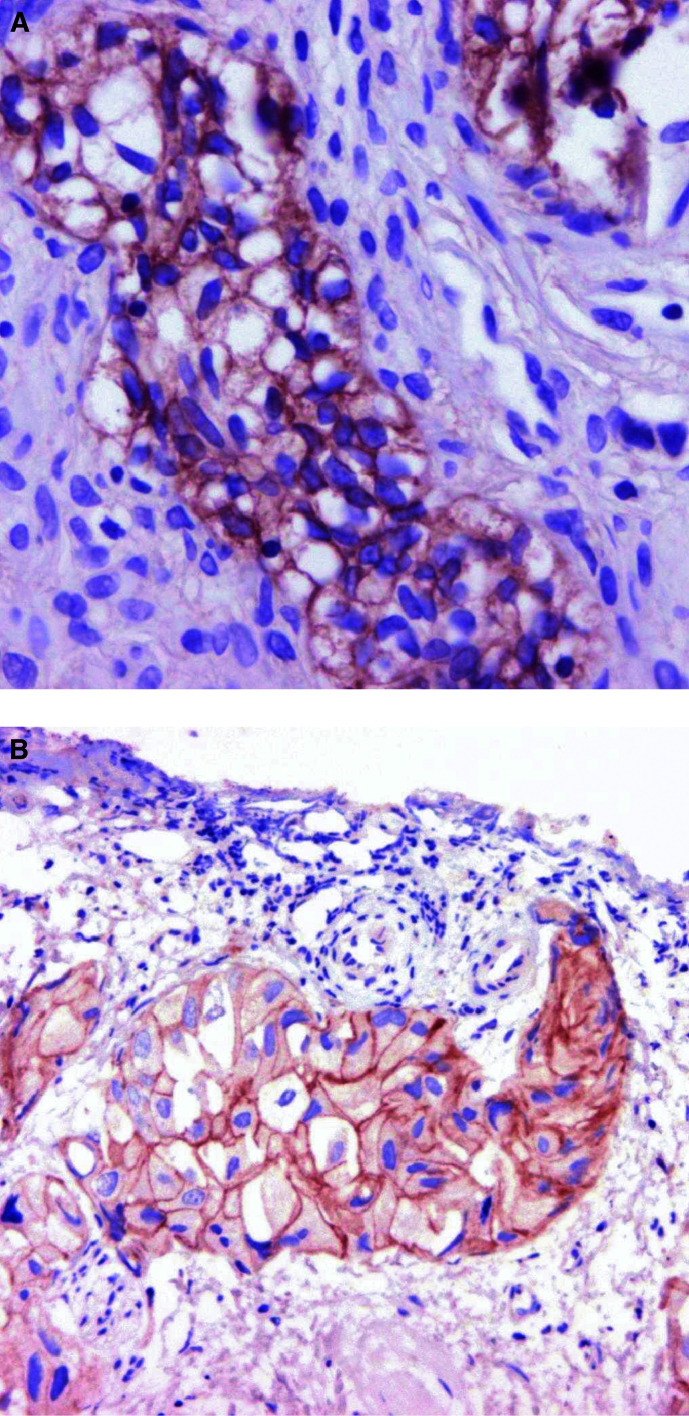
Immunohistochemical detection after the development of AIPC of (**A**) c-erbB-1 (magnification × 40) and (**B**) c-erbB-2 (magnification × 20).

The percentage of PSA immunoreactive specimens decreased from 83% (88 of 106 patients) to 69% (73 patients) as the patients developed AIPC (Table 3). A total of 21 patients lost PSA positivity as they developed AIPC. Before androgen deprivation, c-erbB-1 positivity was demonstrated in 24 patients (23%) and in 46 patients (43%) after the development of AIPC (*P*=0.001). The comparable figures for c-erbB-2 were 33% and 24%, respectively. c-erbB-1 and c-erbB-2 IHC positivity after the development of AIPC is illustrated by Figure 1A–B

.

**Figure 2:**
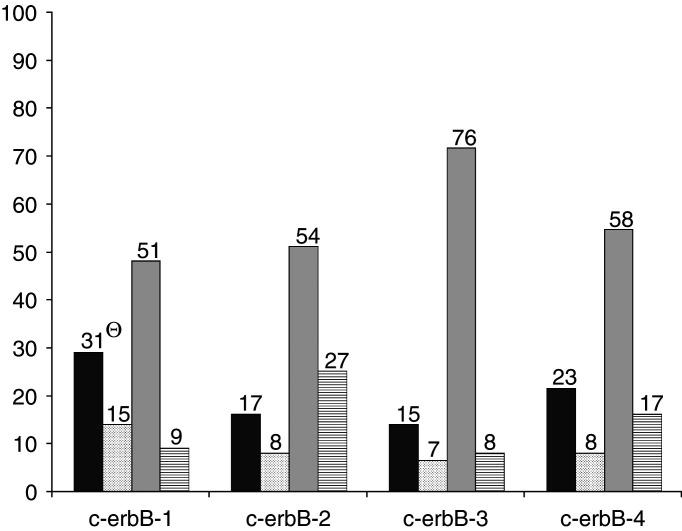
Intrapatient change (PRTR → AIPC) of IHC stainability (106 patients). ▪ Negative → Positive; ▪ Positive → Positive; □ Negative → Negative; 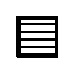 Positive → Negative. Θ Number of patients.

The EGFRs remained negative in 48–71% of the patients during the development of AIPC (Figure 2

). Of 82 patients whose PRTR biopsies were c-erbB-1-negative, 31 displayed c-erbB-1 positivity in their AIPC biopsy (*P*=0.001). However, nine of 24 patients with c-erbB-1 positivity before hormone treatment lost this immunoreactivity after the development of AIPC. The direction of intrapatient changes for c-erbB-2, c-erbB-3 and c-erbB-4 was less pronounced. Overall, the positivity of EGFRs was not significantly related to Gleason score or PSA immunoreactivity (data not shown). During the development of AIPC, the number of c-erbB-1-positive specimens was doubled in those patients who lost PSA positivity, whereas the number of c-erbB-2 positive specimens decreased from eight to three (Table 4

**Table 4:** Growth factor receptors in 21 patients with a PSA-positive PRTR biopsy changing to PSA negativity at the development of AIPC

	**PRTR biopsy**	**AIPC biopsy**	***P*^a^**
*IHC positivity*			
c-erbB-1	6 (29%)	11 (52%)	0.180
c-erbB-2	8 (38%)	3 (14%)	0.125
c-erbB-3	5 (24%)	5 (24%)	1.00
c-erbB-4	7 (33%)	7 (33%)	1.00

a McNemar test for related samples.

). For the 10 specimens of normal prostate tissue, four displayed c-erbB-1 positivity, whereas none were positive for c-erbB-2, c-erbB-3 or c-erbB-4.

### Survival

**Figure 3:**
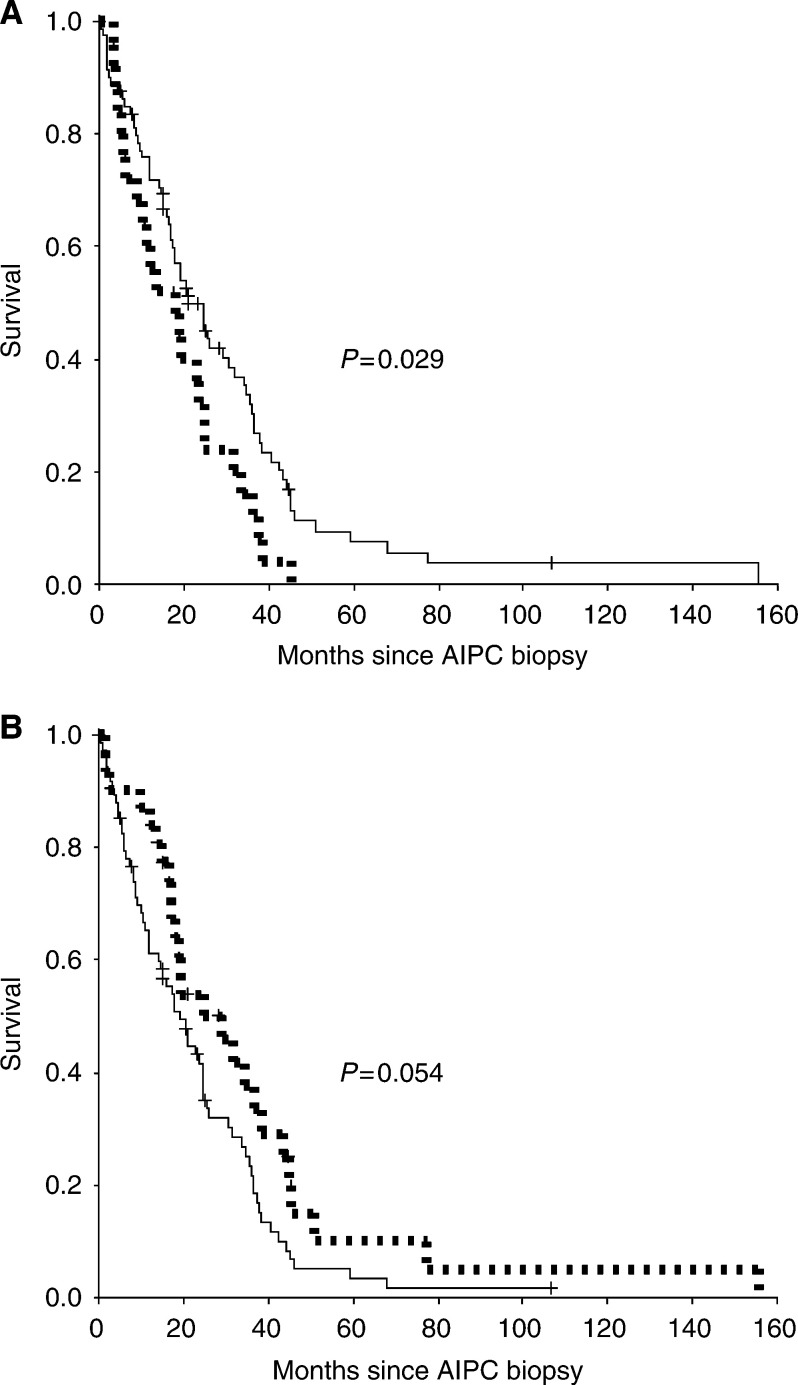
(**A**) C-erbB-2 stainability of the AIPC biopsy and overall survival. — c-erbB-2 negative (81), - - - - c-erbB-2 positive (25). (**B**) C-erbB-4 stainability of the AIPC biopsy and overall survival. — c-erbB-4 negative (75), 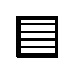 - - - - c-erbB-4 positive (31).

At the time of the last observation, 91 patients (86%) were dead. The median overall survival time from the date of the AIPC biopsy was 20 months (95% CI: 15–26 months). The immunoreactivity of c-erbB-2 determined decreased survival (*P*=0.029) (Figure 3A

), whereas the positivity of c-erbB-4 tended to indicate a better 2-year survival than c-erbB-4 negativity (*P*=0.054) (Figure 3B) (Table 5

**Table 5:** EGFR family expression and survival from the date of AIPC biopsy

**Immunoreactivity**	**Number of patients (%)**	**2-year survival**	***P*^a^**
*c-erbB-1*			
Negative	60 (57%)	0.526	
Positive	46 (43%)	0.362	0.118
			
*c-erbB-2*			
Negative	81 (76%)	0.498	
Positive	25 (24%)	0.320	0.029
			
*c-erbB-3*			
Negative	84 (79%)	0.463	
Positive	22 (21%)	0.409	0.266
			
*c-erbB-4*			
Negative	75 (71%)	0.416	
Positive	31 (29%)	0.539	0.054

a Logrank test for trend.

). The coexpression of c-erbB-1 and c-erbB-2 in the AIPC biopsy (17 patients) did not represent a statistically significant poor prognosis factor (*P*=0.064).

## DISCUSSION

In this descriptive study, 43% of the prostate cancer specimens obtained from growing pelvic tumours of AIPC patients expressed the c-erbB-1 protein and 24% expressed c-erbB-2, the latter being a poor prognosis factor. During the development of AIPC, intrapatient changes occurred in two opposite directions for each of the four EGFRs: (1) negativity of the PRTR biopsies changed to positivity of the paired AIPC biopsies and (2) positivity of the PRTR biopsies changed to negativity of the paired AIPC biopsies. The development of AIPC was significantly associated with the increase of c-erbB-1 expression, whereas no changes for the other growth factor receptors reached the level of statistical significance. The Gleason score and PSA immunoreactivity were not associated with growth factor receptor expression before or after the development of AIPC. In normal prostate tissue, four out of 10 specimens displayed c-erbB-1 positivity, whereas none were positive for c-erbB-2, c-erbB-3 or c-erbB-4.

Previous observations of the expression of c-erbB-1 and c-erbB-2 in prostate cancer tissue and cell lines have demonstrated highly divergent rates. Our results display figures far below the highest reported values of c-erbB-1 and c-erbB-2 expression, but for c-erbB-2 our figures are higher than the results from Savinainen *et al* (2002). The following explanations are offered: Firstly, the cancer cell population in biopsies from human AIPC is much more heterogeneous than that from laboratory cell lines. Secondly, there may be differences in the pattern of EGFRs between AIPC tissue from the prostatic tumour, as used in the present study, and that observed in metastatic cancer tissue as examined by Scher *et al* (1995). Tissue from metastases may express higher levels of growth factor receptors than the primary tumour. Thirdly, several methodological differences as to IHC may lead to variability of the results. In our study, only moderate or strong membrane staining was considered a positive finding, whereas both Scher *et al* (1995) and Di Lorenzo *et al* (2002) included weak membrane staining of c-erbB-1 in their cohort of positive specimens. The use of different antibodies represents another cause of variability of the results. Finally, uncontrollable variations as the duration of formalin fixation of the archival specimens may additionally influence the immunoreactivity of EGFRs.

The results of the present study are in agreement with the published observations: expression of c-erbB-1 increases significantly, as the tumour becomes androgen independent. Xie *et al* (1995) reported that c-erbB-1-mediated signals are associated with the invasiveness of DU-145 human prostate carcinoma cells. However, we were unable to confirm the findings of Di Lorenzo *et al* (2002), that c-erbB-1 expression increased with increasing Gleason score. Unlike the series of Di Lorenzo *et al*, 80% of the untreated cases of the present study were already Gleason score ⩾7b. Di Lorenzo *et al* also found a positive correlation between serum PSA and c-erbB-1, which was not confirmed in our study applying IHC-detected PSA. Lee *et al* (2003) indicated that decreased PSA secretion in androgen-independent LNCaP C-81 cells is associated with a low expression of c-erbB-2, similar to our observation of a trend of reduced c-erbB-2 expression in biopsies that became PSA negative.

Even though high levels of c-erbB-1 can be demonstrated in human prostate cancer, especially after the development of AIPC, its clinical role is not yet clear. This receptor's function is further obscured as it has become clear that internalisation has to take place for its activation (Kim *et al*, 2003). Thus, the protein expression of c-erbB-1 *per se* is only one of several conditions for this receptor's functionality.

The role of c-erbB-2 in prostate cancer is also controversial, contrary to this receptor's clinical importance for advanced breast cancer (Cobleigh *et al*, 1999; Slamon *et al*, 2001). As seen in the present study, immunoreactivity for this receptor has been reported in 9% (Mark *et al*, 1999) up to 30% in untreated patients (20%, Osman *et al*, 2001; 25%, Signoretti *et al*, 2000; 29%, Xie *et al*, 1995; and 30%, Fossa *et al*, 2002). Savinainen *et al* (2002) did not find c-erbB-2 immunoreactivity in any of 54 specimens from untreated patients, nor in 20 lymph node metastases or 50 hormone-refractory tumours. The percentage of c-erbB-2-positive cases among untreated prostate cancer patients thus seems generally lower than in breast cancer. During the development of AIPC, several authors agree that c-erbB-2 positivity increases (Xie *et al*, 1995; Signoretti *et al*, 2000; Osman *et al*, 2001), contrary to our results that showed reversal to c-erbB-2 negativity in 27 out of 35 AIPC patients. The c-erbB-2 immunoreactivity may, however, still be important in the clinical management of a subgroup of these patients, and Moasser *et al* (2001) found that cell lines expressing c-erbB-2 are particularly sensitive to ZD1839. Furthermore, c-erbB-2 and c-erbB-3 frequently act together creating heterodimers. Recently, a naturally occurring inhibitor of c-erbB-3 has been detected (Lee *et al*, 2001), and at least in some tumour cell lines, ZD1839 also inhibits c-erbB-3 (Moasser *et al*, 2001).

Both c-erbB-1 and c-erbB-2 immunoreactivity have been shown to be associated with an unfavourable prognosis in a homogenous series of hormonally untreated cancer patients, in particular if these two receptors are combined (Di Lorenzo *et al*, 2002). Owing to the clinical heterogeneity of our untreated cases, we did not perform a survival analysis based on the PRTR biopsies. However, we found a significant association between c-erbB-2 positivity of AIPC biopsies and a poor prognosis, whereas coexpression of c-erbB-1 and c-erbB-2 in AIPC patients was not statistically significantly associated with an unfavourable prognosis. The finding of a slightly better survival in AIPC patients with c-erbB-4 immunoreactivity needs further confirmation. Previously, the association between c-erbB-4 positivity and a better clinical outcome has been demonstrated for advanced breast cancer patients (Suo and Nesland, 2002).

The strength of the present study is its large number of patients and the possibility to analyse intrapatient changes of expression of EGFRs during the development of AIPC. Our investigations of EGFRs are, however, limited to IHC only. The correlation between IHC-detected c-erbB-2 expression and comparable results of FISH analyses has been debated (Press *et al*, 2002). Analysis by IHC labels the gene product and has been sufficiently accurate for screening purposes, defining positive cases by moderate or strong membrane-specific staining in >10% of the tumour cells. On the other hand, the FISH technique demonstrates gene amplification, and is currently applied in breast cancer patients with nonconclusive c-erbB-2 IHC results and possible therapeutic consequences. It may be discussed whether the pattern of expression of EGFRs in our AIPC patients, all with local problems, is representative for AIPC patients in general. As we have previously indicated, the clinical course and survival of AIPC patients with local problems seem to differ from that of AIPC patients with symptomatic bone metastases (Hernes *et al*, 2000,^2003^).

In summary, we find that during the development of AIPC intrapatient changes occur in two opposite directions for each of the four EGFRs: negativity change to positivity, and vice versa, statistically significant only for the increase of c-erbB-1 expression (*P*=0.001). c-erbB-2 is a poor prognosis factor in AIPC patients with local problems. These findings support ongoing attempts to develop new treatment strategies for subgroups of prostate cancer patients with the inhibition of EGFRs and their signalling pathways. The sole immunohistochemical demonstration of membrane-located EGFRs in biopsies may give some indications for these agents’ efficacy in the individual patient. However, these receptors’ final association with response rates has to be determined in larger series of patients with known growth factor receptor status.
